# Nanostructured selenium-doped biphasic calcium phosphate with in situ incorporation of silver for antibacterial applications

**DOI:** 10.1038/s41598-020-70776-7

**Published:** 2020-08-13

**Authors:** Lei Nie, Mengjuan Hou, Tianwen Wang, Meng Sun, Ruixia Hou

**Affiliations:** 1 College of Life Sciences, Xinyang Normal University (XYNU), Xinyang, 464000 People’s Republic of China; 2 Department of Mechanical Engineering, Member of Flanders Make, KU Leuven (Catholic University of Leuven), 3001 Leuven, Belgium; 3 Medical School of Ningbo University, Ningbo, 315211 People’s Republic of China

**Keywords:** Biomedical materials, Nanoparticles

## Abstract

Selenium-doped nanostructure has been considered as an attractive approach to enhance the antibacterial activity of calcium phosphate (CaP) materials in diverse medical applications. In this study, the selenium-doped biphasic calcium phosphate nanoparticles (SeB-NPs) were first synthesized. Then, silver was in situ incorporated into SeB-NPs to obtain nanostructured composite nanoparticles (_Ag_SeB-NPs). Both SeB-NPs and _Ag_SeB-NPs were characterized by Fourier transform infrared spectroscopy (FT-IR), X-ray diffraction (XRD), ultraviolet–visible spectroscopy (UV–Vis), X-ray photoelectron spectroscopy (XPS), and Raman spectra. The results confirmed that the SeO_3_^2−^ was doped at the PO_4_^3−^ position and silver nanoparticles were deposited on the surface of SeB-NPs. Next, Transmission Electron Microscopy (TEM) analysis displayed that the prepared _Ag_SeB-NPs had a needle-cluster-like morphology. CCK-8 analysis revealed SeB-NPs and _Ag_SeB-NPs had good cytocompatibility with osteoblasts. The antibacterial activity of the prepared _Ag_SeB-NPs was confirmed by using Gram-negative *E. coli* and Gram-positive *S. aureus*. The above results manifested the significance of the final _Ag_SeB-NPs for biomedical applications.

## Introduction

Tissue engineering has been widely used in bone graft and dentistry due to a large number of patients require grafting resulting from congenital conditions, trauma, and tumor resection, and so on^1–3^. Mineralized tissues, such as bone, tooth enamel, dentin, and cementum, are enriched with significant amounts of ionic substitutions, including sodium, potassium, and carbonate groups. These elements can affect the functionality of related tissues^4,5^. Calcium phosphate (CaP) based nanoparticles, including hydroxyapatite (Ca_10_(PO_4_)_6_(OH)_2_, HA), β-tricalcium phosphate (Ca_3_(PO_4_)_2_, β-TCP), tetracalcium phosphate (Ca_4_(PO_4_)_2_O; TTCP), biphasic calcium phosphate (BCP), and so forth, were well-known in implant surgery because of their excellent biocompatibility, biodegradability, and tunable physicochemical properties. Compared to HA or β-TCP, biphasic calcium phosphate (BCP) composed of HA and β-TCP, performed better mechanical properties, higher biological activity, and adjustable degradation rate^6–10^. Bacteria attached to the implanted medical devices or scaffold can cause healthcare-associated infections, such as the formation of biofilm, which further results in local inflammation and infection. Therefore, the scaffold with excellent antibacterial properties provides an overwhelming advantage in the clinic stage^11,12^.

Many metal nanoparticles, such as silver (Ag), zinc oxide (ZnO), and titanium dioxide (TiO_2_), performed strong antibacterial properties and low toxicity towards mammalian cells, some of these nanoparticles have been widely applied in a range of areas^13–20^. Silver (Ag) is stable in the body fluids and can be used in antibacterial implants due to silver can easily binds to bacterial DNA and RNA, resulting in bacteria death^21^. However, the antibacterial effect of nanoparticles was influenced by size, shape, nanostructure, as well as chemical modification. Furthermore, it has been reported that substitution of metal ions in CaP based nanoparticles also showed powerful antibacterial effects, such as selenium-substituted hydroxyapatite (Se-HA) nanoparticles^22^. Se-HA nanoparticles not only displayed excellent biocompatibility but also inhibited specific bacterial strains of interest^23,24^. Matesanz et al*.* demonstrated that the osteoblasts-like cells and preosteoblasts-like cells could adhere to and proliferate on the Se-HA based scaffold, which leads to enhance osteogenesis ability compared to HA-based nanocomposites^25^.

Moreover, selenium (Se) is an essential element for the human body. Adults require 50–70 µg of selenium per day to make selenoproteins, which play a vital role in the human antioxidant defense system and redox control of cellular reactions^26^. Importantly, intaking low selenium increases the risk of mortality, poor immune function, and cognitive decline. Selenium can penetrate bacteria cells, catalyze the oxidation of intracellular thiols, and generate the singlet oxygen, which further causes the death of bacteria^27,28^. Besides bacteria inhibition, selenium doped nanoparticles also show effective anti-cancer properties^29^. However, the synergistic effect of silver and selenium on CaP based nanoparticles in inhibiting bacteria needs to be further investigated.

**Figure 1:**
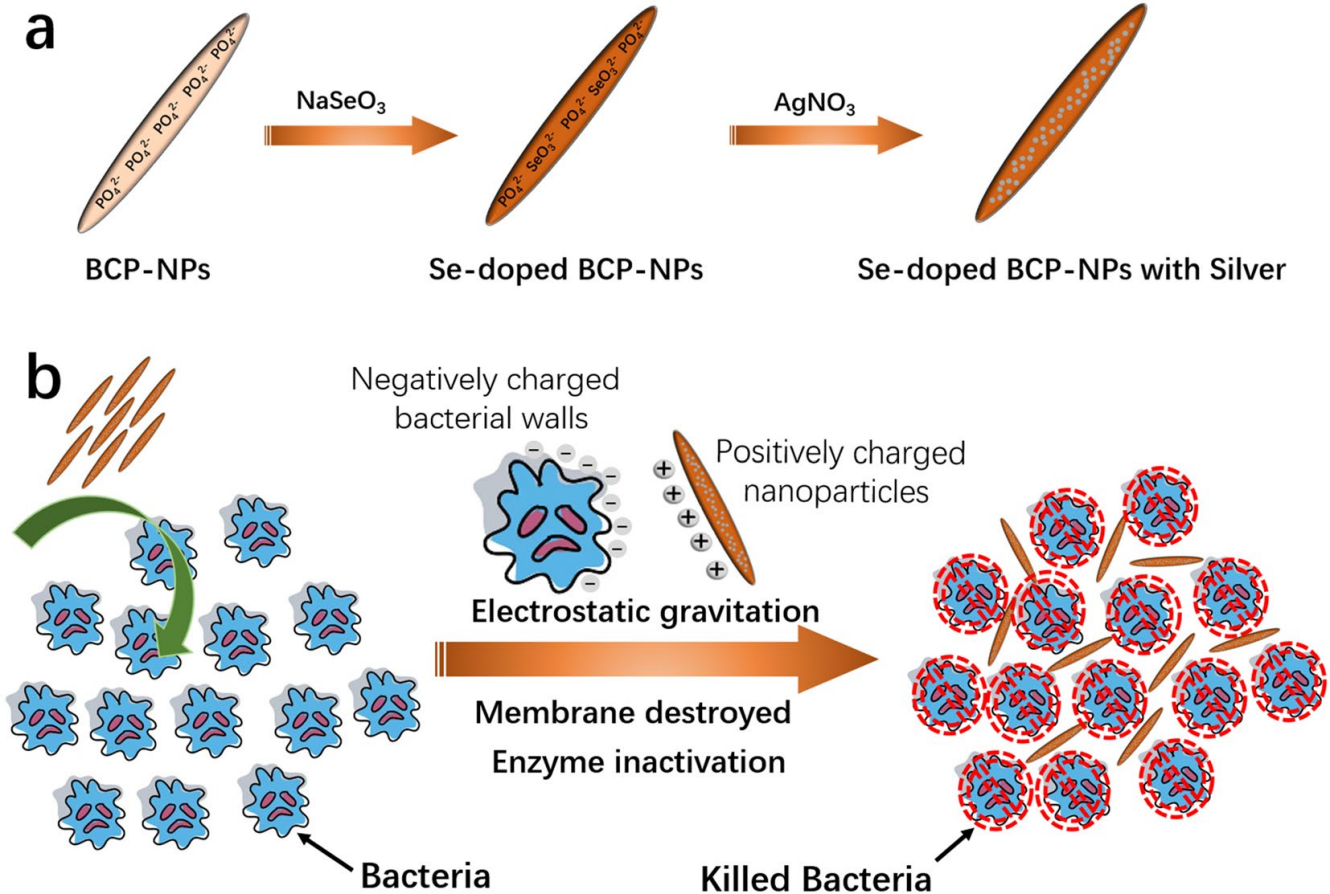
**(a)** Schematic illustration of the preparation of selenium-doped biphasic calcium phosphate nanoparticles with the incorporation of silver (_Ag_SeB-NPs). (**b**) Illustration of the antibacterial mechanism of _Ag_SeB-NPs.

Here, for the first time, the nanostructured selenium-doped BCP nanoparticles with in situ incorporated silver (_Ag_SeB-NPs) were fabricated via an easy and rapid precipitation method. First, selenium-doped BCP nanoparticles (SeB-NPs) were synthesized, and then, silver was deposited on SeB-NPs to obtain _Ag_SeB-NPs (Fig. 1a). The physicochemical properties of the prepared nanoparticles were characterized by Fourier transform infrared spectroscopy (FT-IR), X-ray diffraction (XRD), ultraviolet–visible spectroscopy (UV–Vis), X-ray photoelectron spectroscopy (XPS), Raman spectra, and transmission electron microscopy (TEM). Next, the cytocompatibility of the prepared nanoparticles was evaluated by culturing with osteoblasts. Due to the positively charged _Ag_SeB-NPs nanoparticles (silver ions), the bacteria cell membrane was destroyed, and the enzyme was inactivated until bacteria die, as shown in Fig. 1b. Thus, the antibacterial activity against Gram-negative *E. coli* and Gram-positive *S. aureus* was lastly investigated, and the results proved that the prepared _Ag_SeB-NPs had the potential for antibacterial application in tissue engineering.

## Materials and methods

### Materials

Sodium selenite (Na_2_SeO_3_, 97.0%) was purchased from Shanghai Tyrael Chemical, Co., Ltd. Ammonium phosphate dibasic ((NH_4_)_2_HPO_4_, 99.0%) was obtained from Shanghai Macklin Biochemical Co., Ltd. Calcium nitrate tetrahydrate (Ca(NO_3_)_2_·4H_2_O, 99.0%) was purchased from Sinopharm Chemical Reagent Co., Ltd. Silver nitrate (AgNO3, 99.8%) was obtained from Tianjin Tiangan Chemical Technology Development Co., Ltd. Ammonium solution ( NH_3_·H_2_O, 25–28%) was purchased from Ron Reagent Co., Ltd. Ultrapure water was prepared by a Milli-Q50 SP Reagent Water System (Millipore Corporation, MA, USA). The chemicals used in this study were all analytical grade procured from commercial sources, without further purification.

### Preparation of selenium-doped biphasic calcium phosphate nanoparticles (SeB-NPs)

The SeB-NPs were prepared according to our previous reports with modification^30,31^. Briefly, 50 mL mixed solution of (NH_4_)_2_HPO_4_ and Na_2_SeO_3_ was added in a three-neck flask (different SeB-NPs samples was synthesized mainly depend on adjusting the molar concentration of (NH_4_)_2_HPO_4_ and Na_2_SeO_3_, Electronic Supplementary Material, Table S1), and 50 mL solution of Ca(NO_3_)_2_·4H_2_O (1.69 M) was added, then the pH of mixed solution in the flask was adjusted to 11.0 by adding ammonium solution. The mixed solution was stirred for 24 h, and the precipitates were collected by centrifugation (10,000 rpm) and washed using Millipore water five times. The precipitates were dried at 60 °C for 24 h to obtain SeB-NPs powder. Different SeB-NPs (SB1, SB2, and SB3) were synthesized by adjusting Ca/(P + Se) and Ca/Se mole ratios, as shown in Table 1. At the same time, biphasic calcium phosphate nanoparticles (BCP-NPs) and selenium-doped hydroxyapatite nanoparticles (SeHA-NPs) were prepared for comparison (Electronic Supplementary Material).

**Table 1:** The designation of selenium-doped biphasic calcium phosphate nanoparticles (SeB-NPs).

Nanoparticles	SeB1	SeB2	SeB3
Ca/(P + Se)^a^	1.55	1.55	1.55
Ca/Se^a^	0.05	0.15	0.30

^a^Mole ratio.

### Preparation of SeB-NPs with incorporation of silver (_Ag_SeB-NPs)

200 mg of SB3 nanoparticles was dispersed in 100 mL Millipore water in a three-neck flask, and 30 mg of AgNO_3_ was added into flask slowly. The solution was stirred for 3 h at room temperature (RT). The precipitates were collected by using centrifugation (10,000 rpm) and washed five times using Millipore water to obtain _Ag_SeB-NPs. Different _Ag_SeB-NPs samples were fabricated by regulating the amount of AgNO_3,_ as shown in Table 2.

**Table 2:** The designation of SeB-NPs with the incorporation of silver (_Ag_SeB-NPs).

Nanoparticles	_Ag_SeB1 (mg)	_Ag_SeB2 (mg)	_Ag_SeB3 (mg)
AgNO_3_	30	60	90
SeB3	200	200	200

### Physicochemical characterization of SeB-NPs and _Ag_SeB-NPs

#### Fourier transform infrared spectroscopy (FT-IR) analysis

FT-IR (ThermoFisher, Nicolelis5) was used to confirm the presence of specific chemical groups in SeB-NPs and _Ag_SeB-NPs. The nanoparticles powders were mixed with KBr, ground, and pressed into thin sections, and the KBr was measured as blank control. FT-IR spectra were obtained within the range between 4,000 and 500 cm^−1^ with a resolution of 1 cm^−1^.

#### X-ray diffraction (XRD) analysis

Both SeB-NPs and _Ag_SeB-NPs powders were analyzed by X-ray diffraction (XRD). A X-ray diffractometer (Rigaku Smartlab 9 kW), operating at 45 kV and 200 mA with Cu Kα radiation (λ = 1.5406 Å) and a spinning sample holder, was used to collect the X-ray powder diffraction (XRD) patterns. Data were acquired in the 2θ range of 10°–90° at a step increment of 0.05°.

#### Ultraviolet–visible spectroscopy (UV–Vis) analysis

To investigate the diffuse reflectance spectra of samples, Ultraviolet–visible Spectroscopy (UV–Vis, PerkinElmer, Lambda 950) was used. This instrument was equipped with an integrating sphere attachment. The sample was operated in the range of 200–800 nm at 298 K for the optical diffuse reflectance (DRS) spectra.

#### Dynamic light scattering (DLS) analysis

The size distribution of nanoparticles was examined by Dynamic Light Scattering (DLS, Malvern Zetasizer 3000E). Zeta potential measurements were performed by Laser Doppler Anemometry with a Zetasizer Nano ZS/Masterszer 3000E. Electrophoretic mobility (converted into ζ-potential by the Smoluckowsky approximation) of nanoparticles was tested at 25 °C.

#### Morphology analysis

The morphology of SeB-NPs and _Ag_SeB-NPs was investigated by Transmission Electron Microscopy (TEM, Tecnai G2 F20) with an energy-dispersive detector (EDS) and Elemental Mapping accessories. The obtained nanoparticles were dispersed in ethanol and sonicated for 2 h, and then the copper grid was dipped into a sample solution and dried under an infrared lamp. Furthermore, the morphology of SeB-NPs and _Ag_SeB-NPs were observed with a cold field emission scanning electron microscope (SEM, Hitachi, S-4800). Before SEM observation, the samples were coated with a thin Pt conductive layer, energy dispersive X-ray Spectroscopy (EDX) was used for the elemental composition analysis or chemical characterization.

#### Raman spectroscopy analysis

Raman spectra of both SeB-NPs and _Ag_SeB-NPs were recorded with a Spex Model spectrometer (LabRAM HR) from 200 to 1,600 cm^−1^ using the 488 nm wavelength excitation from an argon-ion laser.

#### X-ray photoelectron spectrometer (XPS) analysis

Both SeB-NPs and _Ag_SeB-NPs were also measured by X-ray Photoelectron Spectrometer (K-Alpha 0.05 eV, Thermo Scientific) to obtain their elements composition, the detailed sample preparation for XPS test refers to our previous paper^6^.

### Cell culture and cytocompatibility of SeB-NPs and _Ag_SeB-NPs

Regarding the potential bone tissue engineering application in the future, hFOB 1.19 cell (osteoblast type, ATCC® CRL-11372™) was used to evaluate the cytocompatibility of SeB-NPs and _Ag_SeB-NPs. According to ATCC protocols, hFOB cells were cultured using Dulbecco’s modified Eagle’s medium (DMEM) (Sigma-Aldrich) supplemented with 10% fetal bovine serum, 100 U mL^−1^ penicillin, and 100 μg mL^−1^ streptomycin. Cells were grown in the tissue culture flask (50 mL) under a humidified atmosphere of 95% air and 5% CO_2_ at 37 °C. The culture medium was changed every 2 days. The cells were passaged by trypsinization, and cells at passage 5 were used for the next experiments. First, a certain amount of obtained SeB-NPs and _Ag_SeB-NPs were added into the cell medium, and no agglomeration phenomenon was not observed under optical microscopy. The cell viability cultured with nanoparticles was quantitatively investigated by the Cell Counting Kit-8 (CCK-8, Abcam) assay, and the CCK-8 assay was operated according to the CCK-8 Cell Proliferation Assay Kit protocol. Finally, the absorbance at 450 nm was measured by using a microplate reader to indicate cell proliferation after culturing with nanoparticles (Electronic Supplementary Material).

### Antibacterial activity assay

Gram-negative *E. coli* (ATCC 25922) and Gram-positive *S. aureus* (ATCC 6538) were used to evaluate the antibacterial activity of prepared SeB-NPs and _Ag_SeB-NPs. The single colony of *E. coli* and *S. aureus* on the Luria Bertani (LB) agar plate were transferred to a liquid LB culture medium by growing at 37 °C overnight to obtain seed culture. 100 μL of nanoparticles dispersed in ultrapure water (the concentrations of nanoparticles used were 500 μg/mL, 1,000 μg/mL and 2000 μg/mL, respectively) were mixed with 10 mL autoclaved LB medium, then 5 μL of seed cultures of *E. coli* or *S. aureus* was inoculated into the medium. After culturing for 12 h at 37 °C, the optical density at 600 nm (OD_600_) was tested by a UV–Vis spectrophotometer. *E. coli* and *S. aureus* were grown at the same conditions without adding nanoparticles as the control group. On the other hand, the seed culture medium was diluted into fresh LB medium and cultured under 37 °C. When the OD_600_ of medium reached about 0.6, the broth was diluted to 105 CFU mL^−1^ with sterile 0.9% NaCl solution. After then, the suspension (50 μL) was spread onto a 90 mm-diameter LB agar plate. The wells were created with a hole puncher with a diameter of 4 mm. 30 μL of the prepared nanoparticles solutions (500 μg/mL, 1,000 μg/mL and 2000 μg/mL) were added into the wells. Then, the plates were kept in an incubator at 37 °C for 12 h, the inhibition zones for each sample were recorded.

### Statistics

All data were expressed as means with standard deviation. SPSS software (SPSS Inc, Chicago IL) was used for the analysis. Statistical analyses were performed by ANOVA or 2-way repeated-measures ANOVA with Tukey’s test applied to investigate specific differences. Statistical significance was defined at a *p *value of < 0.05 for 95% confidence.

## Results

### Physicochemical characterization

**Figure 2:**
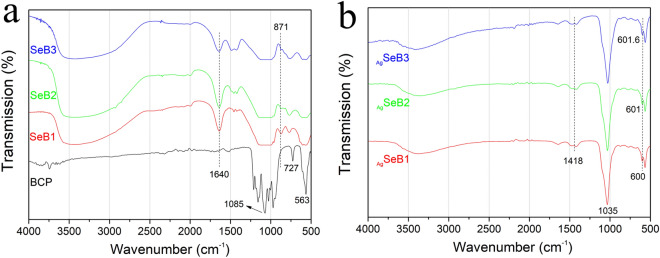
(**a**) FT-IR spectra of BCP-NPs and SeB-NPs, and (**b**) FT-IR spectra of _Ag_SeB-NPs.

First, the chemical groups were analyzed by FT-IR spectroscopy, and the spectra of SeB-NPs and _Ag_SeB-NPs were presented in Fig. 2. The broad peak in the range of 3,000–3,800 cm^−1^ was due to H_2_O stretching vibrations in absorbed water. As shown in Fig. 2a, the band at 3,572 cm^−1^ was arisen from the stretching of OH^−^ ions. The bands for BCP at 1,107, 1,136, and 962 cm^−1^ corresponded to the P–O stretching vibration modes of PO_4_^3−^, while for SeB-NPs it decreased and disappeared. At the same time, the band at 563 cm^−1^ was attributed to O–P–O vibration mode, and the band decreased as the Ca/Se ratio increased. Notably, the sharpness of 563 cm^−1^ bands indicated the well-crystallized BCP-NPs, and the peak became rounded, indicated the decrease of crystallization for Se-NPs. However, due to the incorporation of selenium, a band of SeB-NPs at 871 cm^−1^ appeared, which was assigned to SeO_3_^2−^ stretching. The spectra of _Ag_SeB-NPs were shown in Fig. 2b. It was clearly noted that the peak at 1,035 cm^−1^ was ascribed to antisymmetric (*v*^3^) P-O stretching vibration mode, which was mainly due to the combination of the silver nanoparticles with OH^−^ or PO_4_^3−^ groups of SeB-NPs. The slight difference between _Ag_SeB1, _Ag_SeB2, and _Ag_SeB3 demonstrated that the chemical bonding between silver and SeB-NPs barely happened, and silver was mainly deposited on SeB-NPs via electrostatic attraction. Complementary to the above FT-IR analysis, the Raman spectra of both SeB-NPs and _Ag_SeB-NPs were obtained.

**Figure 3:**
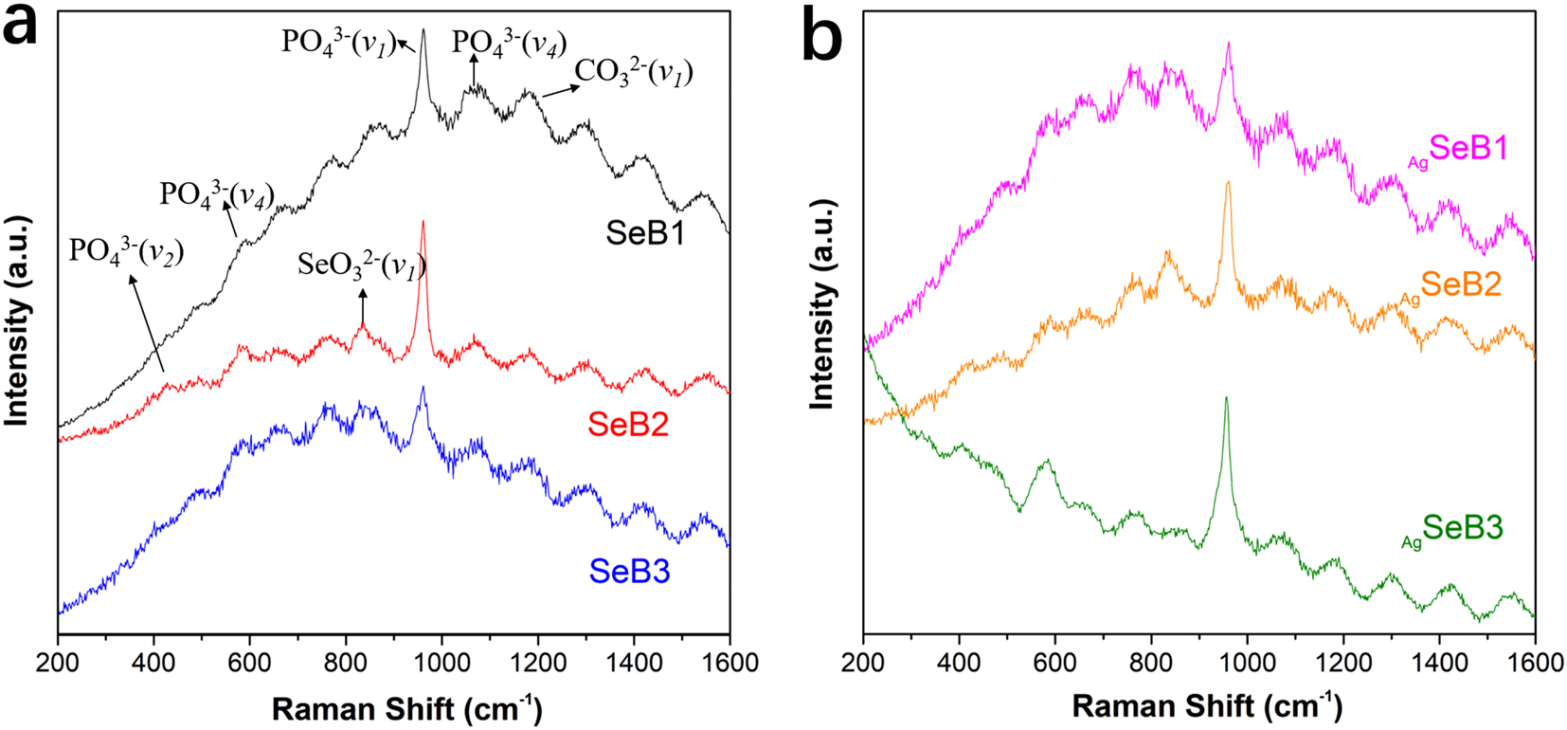
Raman spectra of (**a**) SeB-NPs and (**b**) _Ag_SeB-NPs.

The Raman spectra of SeB-NPs and _Ag_SeB-NPs were shown in Fig. 3. The OH^−1^ vibrational bands in the region of 630 cm^−1^ were not clearly observed, which was in good accord with the FT-IR results. In the case of SeB-NPs, an intense peak appeared at 960 cm^−1^ due to the stretching mode (*v*_1_) of PO_4_^3−^ group, and peaks at 430 cm^−1^, 590 cm^−1^, and 1,070 cm^−1^ were attributed to the stretching mode (*v*_2_), bending mode (*v*_4_), and stretching mode (*v*_3_) of the PO_4_^3−^ group, respectively. The peak at 1,064 cm^−1^ corresponded to the symmetrical stretching vibration mode (*v*_1_) of CO_3_^2−^ group. In addition, the band near 830 cm^−1^ was attributed to the symmetrical stretching mode (*v*_1_) of the SeO_3_^2−^ group. For _Ag_SeB-NPs, similar chemical groups from SeB-NPs appeared. Compared with FTIR spectra, water vibrational modes should give rise to weak intensity stretching and no bending bands in Raman spectra were observed. However, the intensity of the vibration peak at 589 cm^−1^ increased with increasing silver content.

**Figure 4:**
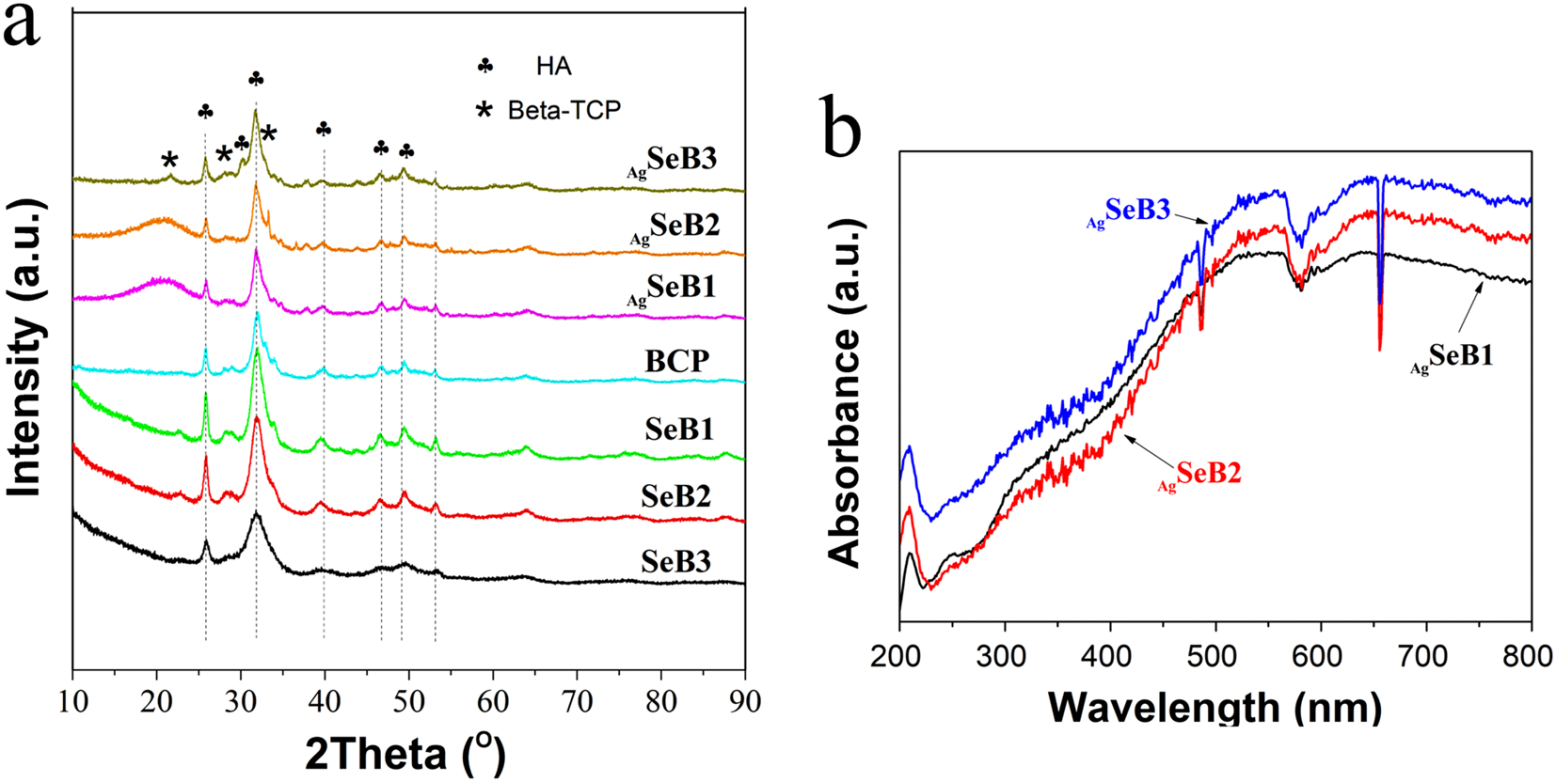
(**a**) XRD spectra of BCP-NPs, SeB-NPs, and _Ag_SeB-NPs, and (**b**) UV–Vis DRS spectra of _Ag_SeB-NPs.

XRD was used to identify the crystalline phase, and the XRD patterns of all of the synthesized samples were shown in Fig. 4a. The peaks of HA and β-TCP were present in BCP, SeB-NPs, and _Ag_SeB-NPs, the peaks at 25.9° (0 0 2), 28.2° (1 0 2), 31.8° (2 1 1), 32.3° (1 1 2), and 49.6° (2 1 3) matched with the standard XRD spectrum of HA (JCPDS No. 9-0432), and the peaks at 22.9° (3 3 -2), 25.9° (1 0 10), 28° (2 1 4), and 31.4° (2 1 10) matched with β-TCP (JCPDS, No. 9-0169). The peaks in the (0 0 2) and (2 1 1) planes were broadened for SeB-NPs, indicating that selenium might enter into the crystal lattice of BCP-NPs and affect the crystallinity of BCP-NPs. Furthermore, the broadening of reflection peaks in XRD patterns for SeB-NPs indicated that the decrease of crystallinity with the increase of Ca/Se ratios, which may be due to the substitution of PO_4_^3−^ by SeO_3_^2−^ group. With increasing selenium content into BCP-NPs, the crystallinity of SeB-NPs decreased. For _Ag_SeB-NPs, no other crystalline phases were detected based on SeB-NPs. The reduced intensity indicated that the crystallinity further decreased gradually with increasing silver content. It was notable that new peaks (2*θ* = 35–40°) were emerged _Ag_SeB-NPs compared to SeB-NPs, and Bragg reflections corresponding to silver were detected (2*θ* = 38.2° and 44.4°, JCPDS, No. 87-0720, Supplementary Materials).

**Figure 5:**
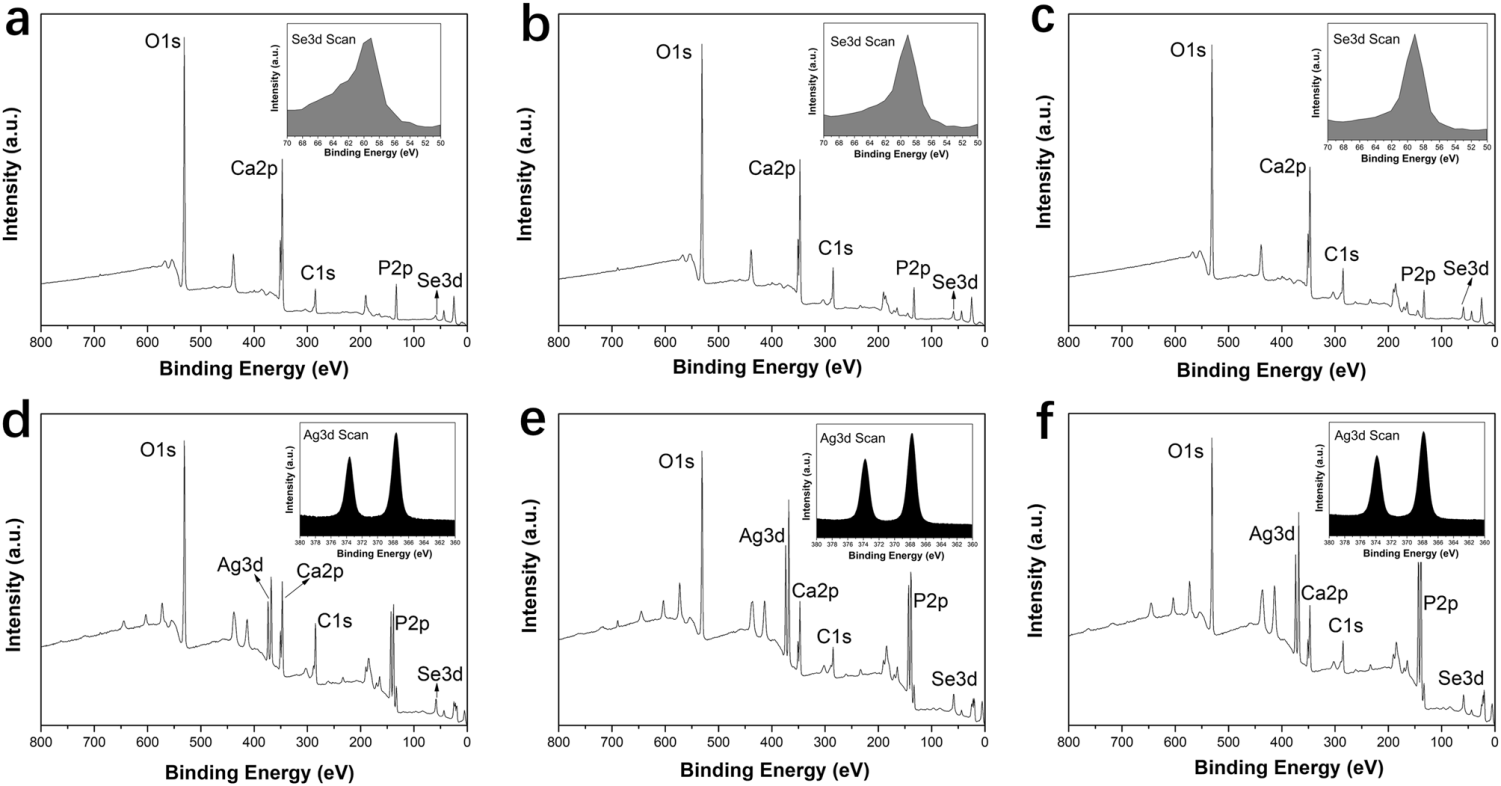
XPS spectra of SeB-NPs, and _Ag_SeB-NPs, (**a**): SeB1; (**b**): SeB2; (**c**): SeB3; (**d**): _Ag_SeB1; (**e**): _Ag_SeB2; (**f**): _Ag_SeB3. The insets for SeB-NPs in the upper row showed the Se3d scanning, and the insets for _Ag_SeB-NPs in the lower row showed the Ag3d scanning.

Furthermore, the UV–Vis DRS spectroscopy was used to analyze the obtained _Ag_SeB-NPs, as shown in Fig. 4b. The UV–Vis DRS absorption broadened in the region of 250–800 nm, and the band at 550 nm corresponds to the surface plasmon resonance of silver nanoparticles. This phenomenon indicated that silver was deposited uniformly on SeB-NPs. To get a further detailed vision for the ionic species of SeB-NPs and _Ag_SeB-NPs, XPS measurements were performed, as shown in Fig. 5. The XPS spectra of SeB-NPs (Fig. 5a–c) found all the expected elements during the preparation process, including Ca, O, C, P, Se. The peak at 58 eV corresponded to SeO_3_^2−^ [Se (IV)] indicated that SeO_3_^2−^ group was already incorporated in BCP-NPs lattice. And the peak at 55 eV belonged to Se (0) was not observed, proving that the redox reaction happened during the preparation process. The P2p spectra of all samples exhibited a peak at 132 eV, which was assigned to the phosphate group. The double peaks at 347 eV and 351 eV were attributed to the Ca2p_3/2_ and Ca2p_1/2_. For _Ag_SeB-NPs, except for the peaks appeared in SeB-NPs, the new high peaks around 367 eV and 373 eV were assigned to Ag3d_5/2_ and Ag3d_3/2_ binding energies, respectively^32,33^.

**Figure 6:**
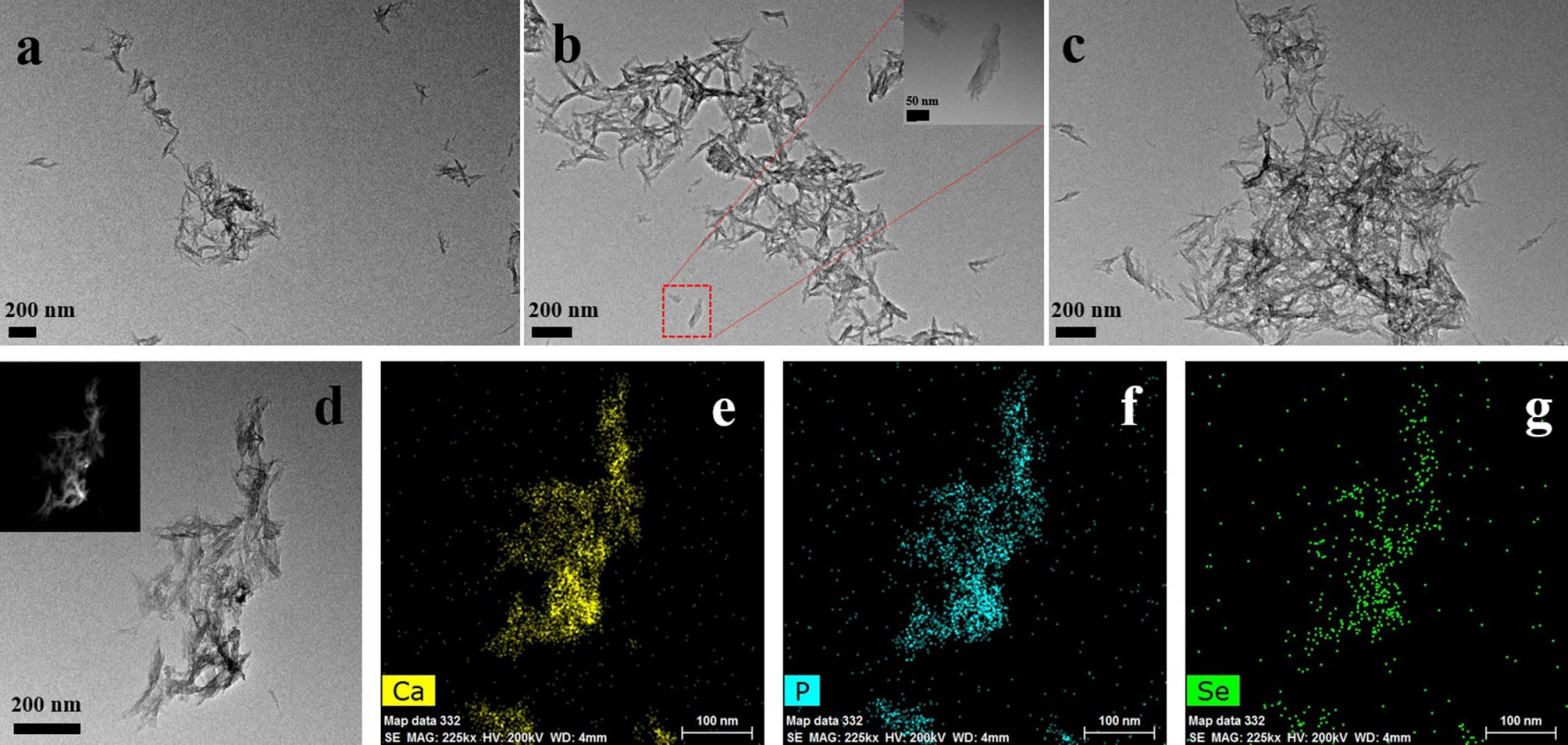
TEM images of the prepared SeB-NPs, (**a**): SeB1, (**b**): SeB2, (**c**): SeB3, the image inserted at the top-right corner of the image (**b**) was enlarged to a higher magnification. Elemental mapping of SeB2 (**d**) revealed the presence of calcium (**e**), phosphate (**f**), and selenium (**g**), the image inserted at the top-left corner of the image (**d**) was acquired High-angle annular dark-field (HAADF) image.

The morphology of the prepared SeB-NPs and _Ag_SeB-NPs was analyzed by TEM. TEM micrographs of SeB-NPs were given in Fig. 6. BCP-NPs exhibited an ellipsoidal morphology (Figure S1, Supplementary Materials). SeB-NPs showed clear needle granular morphology with a length of less than 100 nm. The results revealed that the doping selenium influenced the morphology of SeB-NPs. Agglomeration was observed in all SeB-NPs samples. SEM was used to further confirm the morphology of SeB-NPs, and the SEM images were shown in Figure S2a–f (Supplementary Materials), and the nanoparticles of all samples were aggregated. With increased Ca/Se mole ratio, more SeB-NPs nanoparticles aggregated, which was certified via DLS analysis (Figure S3, Supplementary Materials). Furthermore, the high-angle annular dark-field (HAADF) mode image of sample SeB2 was present in Fig. 6d, which was inserted in the top-left corner, and the elemental mapping results were shown in Fig. 6e–g. The presence of calcium (yellow), phosphorus (blue), and selenium (green) indicated that selenium was evenly distributed in the SeB-NPs.

**Figure 7:**
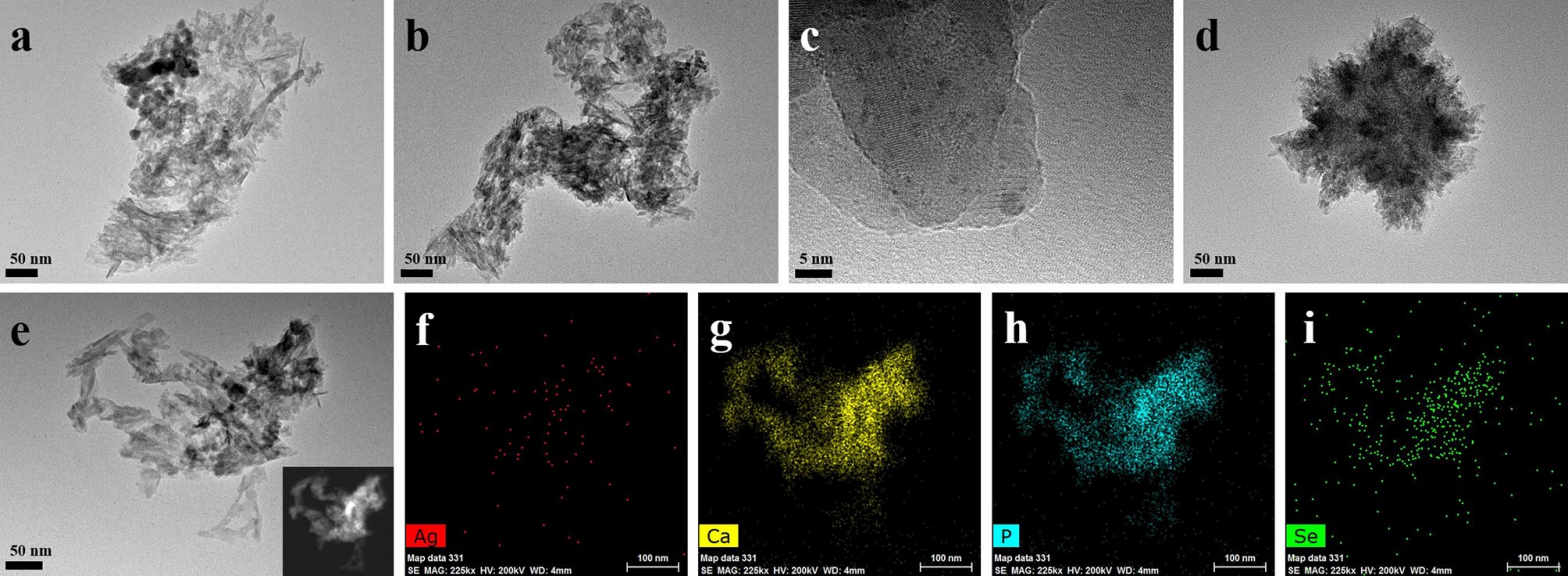
TEM images of the prepared _Ag_SeB-NPs, (**a**): _Ag_SeB1, (**b**, **c**, **e**): _Ag_SeB2, (**d**): _Ag_SeB3, the image (**c**) was enlarged from image (**b**) at a higher magnification. Elemental mapping of _Ag_SeB2 (**e**) confirmed the presence of silver (**f**), calcium (**g**), phosphate (**h**), and selenium (**i**), the image inserted at the bottom-right corner of the image (**e**) was HAADF image.

The TEM images of _Ag_SeB-NPs were displayed in Fig. 7. Compared with SeB-NPs, the _Ag_SeB-NPs displayed irregular morphology, and the aggregation was observed, indicating that the morphology of SeB-NPs was influenced by the deposition of silver nanoparticles. According to the DLS test results (Figure S3c, Supplementary Materials), multiple peaks appeared and confirmed that _Ag_SeB-NPs nanoparticles were further aggregated compared to SeB-NPs, which was also affirmed by SEM (Figure S2g–i, Supplementary Materials). With the increase of AgNO_3_ used during the preparation procedure, the prepared _Ag_SeB-NPs tended to flock together, and the snowflake-like morphology was shown (Fig. 7d). The high-magnification image of _Ag_SeB2 revealed that the silver nanoparticles adhered to the surface of _Ag_SeB-NPs (Fig. 7c). Interestingly, a rooster-like image (like the map of China) was obtained for _Ag_SeB2 sample, as shown in Fig. 7e, and the HAADF mode image was inserted in the bottom-right corner. The elemental mapping analysis showed that silver (red, Fig. 7f), calcium (yellow, Fig. 7g), phosphorus (blue, Fig. 7h), and selenium (green, Fig. 7i) were detected, proved that silver and selenium were evenly distributed in _Ag_SeB-NPs. In addition, EDX analysis was used to further determine the elemental composition of SeB-NPs and _Ag_SeB-NPs, as shown in Figure S4 (Supplementary Materials). According to the EDX analysis of SeB-NPs, the elements oxygen (O), phosphorous (P), calcium (Ca), and selenium (Se) were presented. With increasing Ca/Se mole ratio, the weight percent of selenium calculated from EDX decreased. The weight percentage of silver were 0.06, 0.13, and 0.33 for _Ag_SeB1, _Ag_SeB2, and _Ag_SeB3, respectively, which was consistent with the amount of AgNO3 added during the preparation of _Ag_SeB-NPs.

### In vitro cytocompatibility

**Figure 8:**
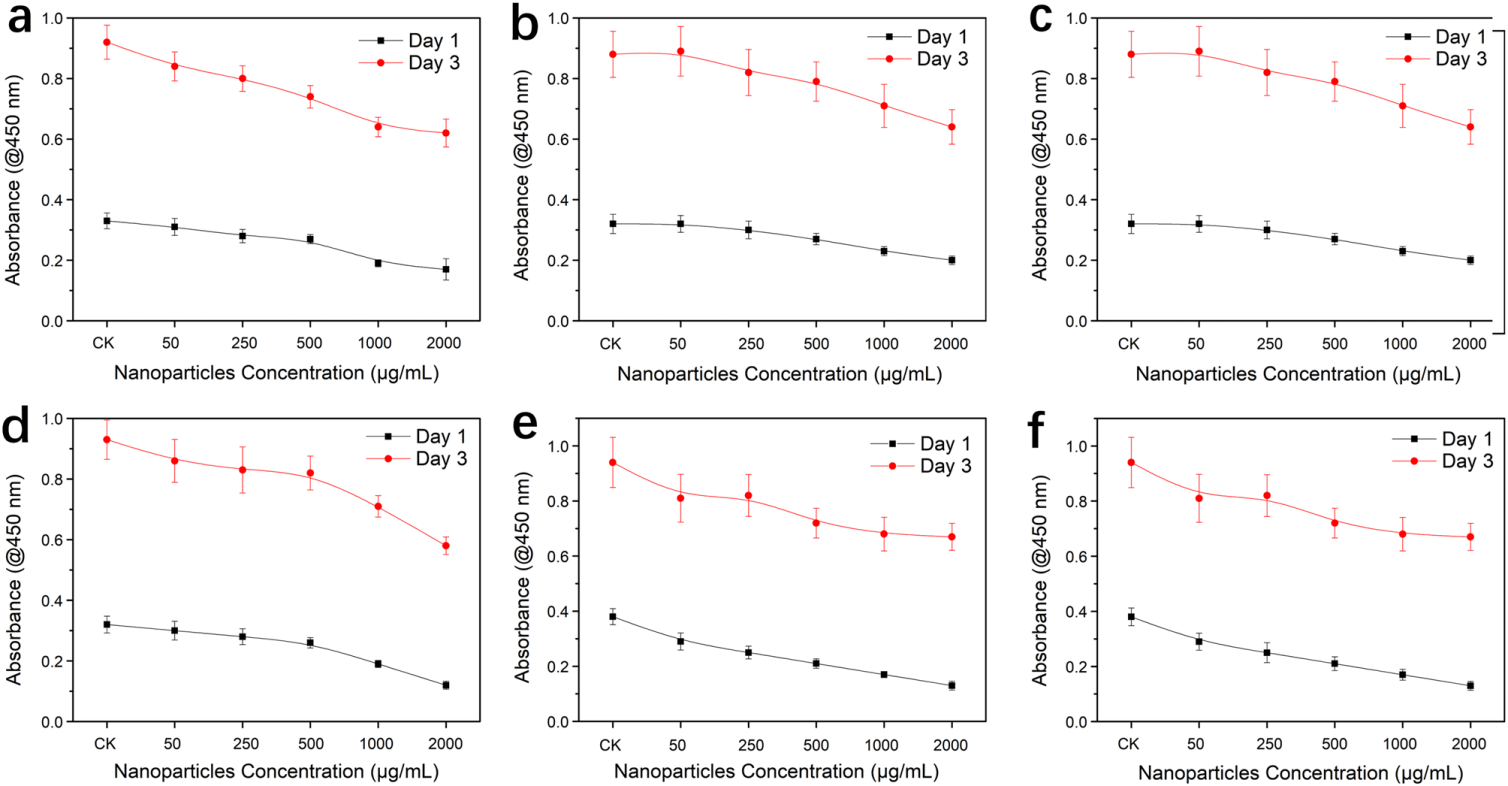
Cell viability (absorbance at 450 nm) of hFOB 1.19 cells exposed to SeB-NPs and _Ag_SB-NPs using different concentrations, which was measured by the CCK-8 assay, (**a**): SeB1; (**b**): SeB2; (**c**): SeB3; (**d**): _Ag_SeB1; (**e**): _Ag_SeB2; (**f**): _Ag_SeB3. Without adding nanoparticles as a control group (CK).

The in vitro cytocompatibility test of the prepared SeB-NPs and _Ag_SeB-NPs is an essential prerequisite for future bone tissue engineering, and biological experiments were conducted by using hFOB 1.19 cell as the cell model. Figure 8 showed the CCK-8 assay results after the hFOB 1.19 cells cultured with SeB-NPs and _Ag_SeB-NPs for 1 and 3 days. The degree of hFOB 1.19 cells growth was recorded by the absorbance at 450 nm. For all prepared nanoparticles, the number of cells increased with the number of culture days indicated that the obtained nanoparticles had an excellent cytocompatibility. However, the cell number decreased with increasing the concentration of nanoparticles from 50 µg/mL to 2000 µg/mL. The cell morphology on day 3 was investigated using optical microscopy and fluorescent microscopy (FITC-Phalloidin/DAPI staining) and 2000 µg/mL of nanoparticles were used (Figures S5 and S6, Supplementary Materials). The cells incubated with the prepared nanoparticles showed a well-preserved morphology, which was polygonal and fully spread. Moreover, the cells proliferated with _Ag_SeB-NPs faster, than that with SeB-NPs. There were no statistically significant differences in cell proliferation culturing with different samples.

### Antibacterial test

**Figure 9:**
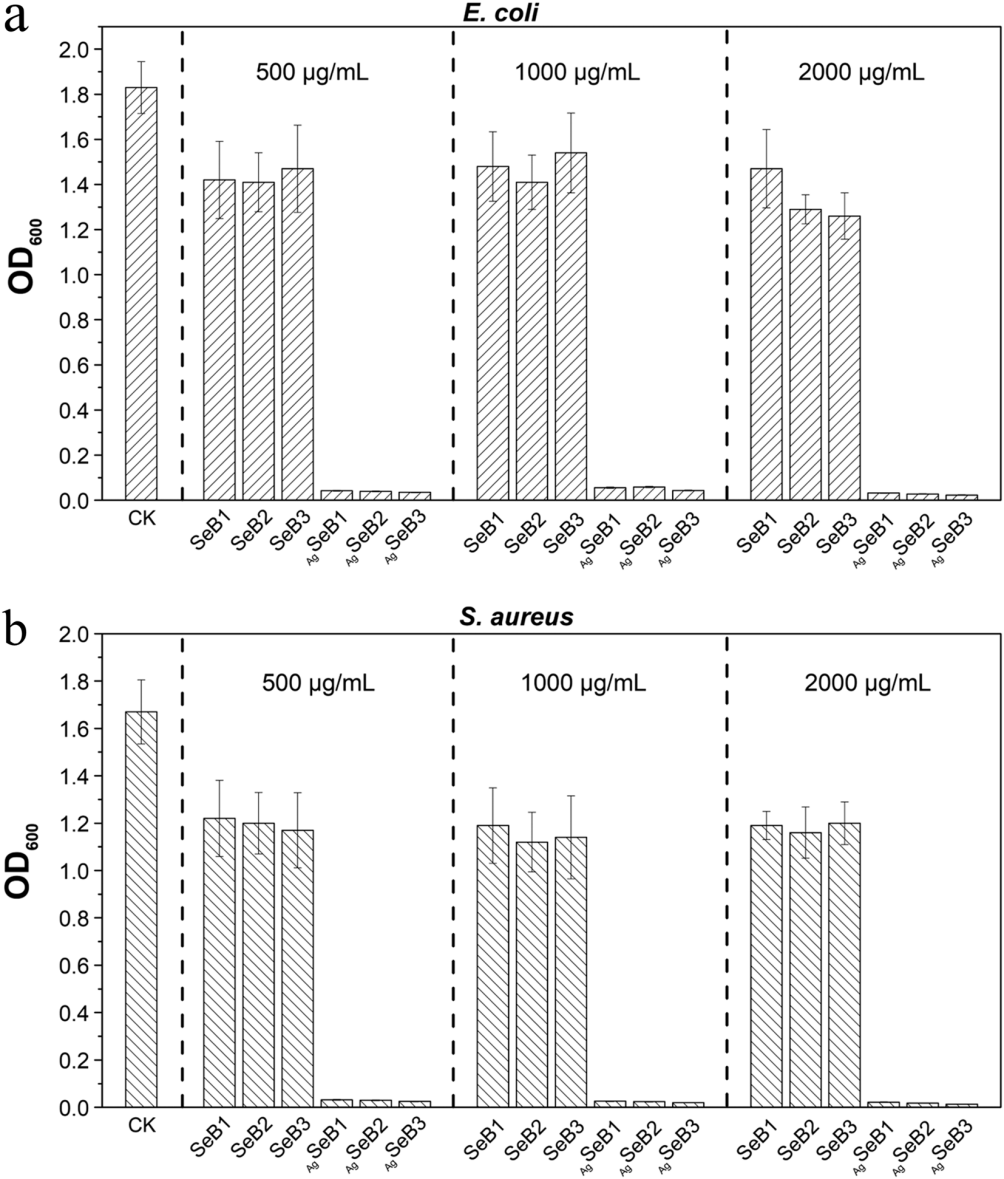
Optical density at 600 nm (OD_600_) of cultured *E. coli* (**a**) and *S. aureus* (**b**) in the LB medium after 12 h supplemented with SeB-NPs and _Ag_SeB-NPs using different nanoparticles concentrations. Without adding nanoparticles as CK group.

**Figure 10:**
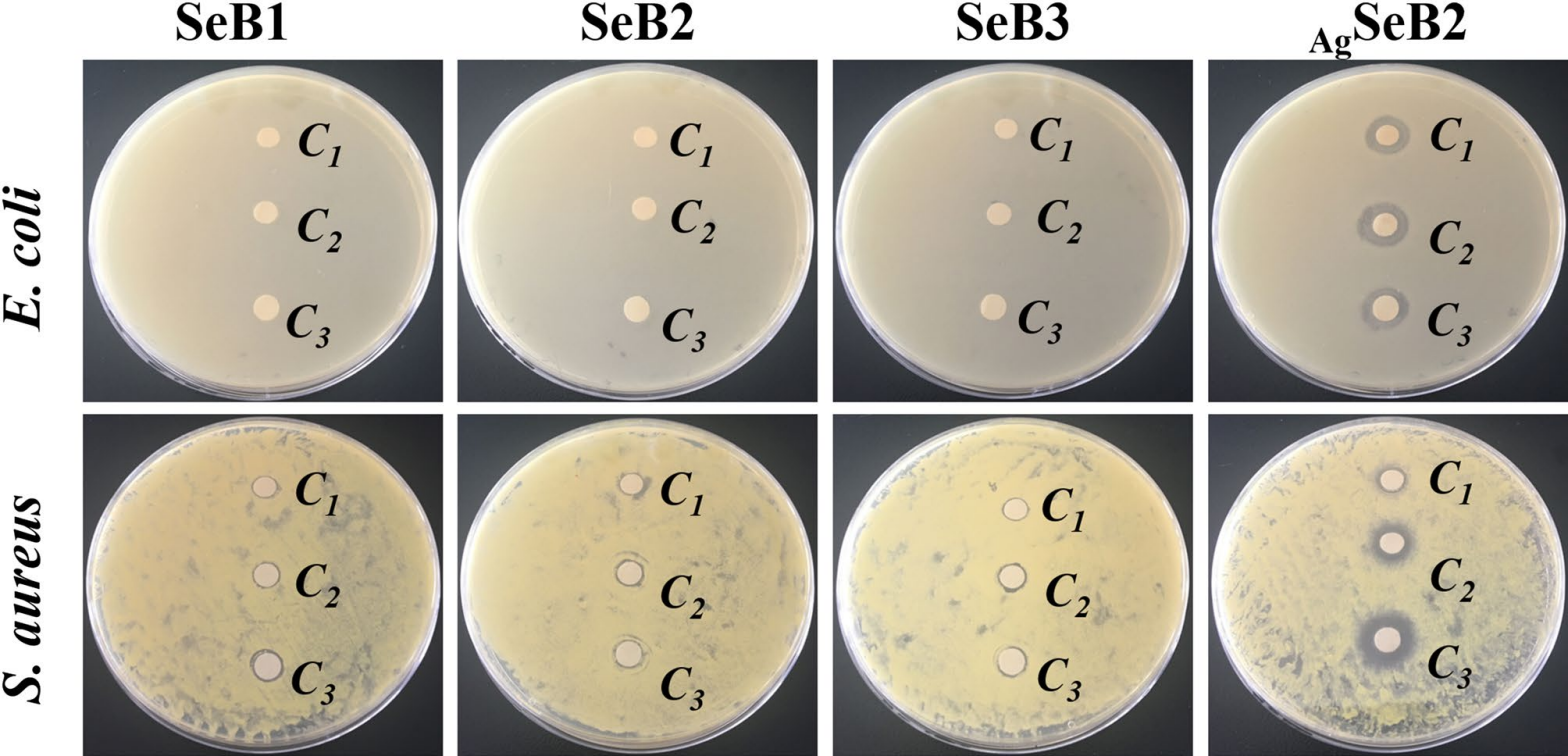
Photos of *E. coli* and *S. aureus* grew on nutrient agar LB plates after the addition of SeB-NPs and _Ag_SeB-NPs for 12 h, C_1_: 500 μg/mL; C_2_: 1,000 μg/mL; C_3_: 2000 μg/mL.

The antibacterial activity of the prepared SeB-NPs and _Ag_SeB-NPs against Gram-positive *S. aureus* and Gram-negative *E. coli* bacteria was systematically evaluated, and the results were shown in Figs. 9 and 10. *S. aureus* could cause the formation of biofilm on bone implants, and *E. coli* strains possess the reduction capability of selenite. Thus, both bacteria were used here. The optical density at 600 nm (OD_600_) values of *S. aureus* and *E. coli* by adding SeB-NPs and _Ag_SeB-NPs using different concentrations were presented in Fig. 9. Compared with the control group, the doping selenium into BCP-NPs had slight influence on the antibacterial activity, and it had a slight inhibitory effect against bacteria of SeB-NPs after 12 h of culture. However, the proliferation of both *S. aureus* and *E. coli* was completely suppressed by _Ag_SeB-NPs. The photos and inhibition ratio of *E. coli* and *S. aureus* grown in the actual culture tubes after adding SeB-NPs and _Ag_SeB-NPs after 12 h were shown in Figures S7 and S8 (Supplementary Materials). Furthermore, the OD_600_ values of *S. aureus* after SeB-NPs were lower than that of *E. coli*, indicated that SeB-NPs had a better antibacterial effect against *S. aureus* than *E. coli*. For _Ag_SeB-NPs, the inhibition ratio of both *S. aureus* and *E. coli* increased with the increase of the AgNO_3_ dosage. There were no differences in _Ag_SeB-NPs against both bacteria. The antibacterial activity of nanoparticles was also confirmed by bacteriostatic circles investigation as shown in Fig. 10.

The disk diffusion was used to confirm the antibacterial effect of prepared SeB-NPs and _Ag_SeB-NPs dispersed in ultrapure water against bacterial colonies. Photos of bacteriostatic circles for nanoparticles using different concentrations were recorded, as shown in Fig. 10. For SeB-NPs, the bacteriostatic circles of *E. coli* were not apparently observed, and such circles of *S. aureus* appeared, in which the concentration of nanoparticles did not have a significant influence on the diameter of circles. However, compared with SeB-NPs, _Ag_SeB-NPs have significant inhibition circles against *S. aureus* and *E. coli*. The antibacterial activity of _Ag_SeB-NPs against *E. coli* was not influenced by the concentration. But, such activity against *S. aureus* increased with increasing of _Ag_SeB-NPs concentration, demonstrated a dose-dependent manner. Furthermore, both SeB-NPs and _Ag_SeB-NPs displayed better antibacterial properties compared to selenium-doped hydroxyapatite (SeHA-NPs) (Figures S9and S10, Supplementary Materials).

## Discussion

The stated objective of this study was to synthesis new-style biphasic calcium phosphate nanoparticles (BCP-NPs) with excellent cytocompatibility and antibacterial activity for further hard-tissue engineering applications. Recently, silicon, silver, copper, zinc, selenium, iron, lithium, and titanium dioxide, et al*.,* have been employed to mingle with bone scaffolds to improve their physicochemical properties^34–40^. Such as, silicon could be doped into hydroxyapatite and composited into gelatine, and then, the three-dimensional (3D) printable composites were obtained. The porous scaffold could be fabricated by rapid prototyping at room temperature (RT)^39^. The bone regeneration of bioactive silicate glass could be improved when the copper was doped at a controlled concentration (0–0.8 wt.% copper oxide), while displayed a promising antibacterial activity^41^. Furthermore, compared with pure hydroxyapatite (HA) scaffold, lithium-doped hydroxyapatite scaffold not only showed a higher degradation rate but also benefit the proliferation of osteoblasts^42^. Ahmed et al*.* confirmed that the introduction of selenium into carbonated hydroxyapatite (CHAP) could increase the diffusion and infiltration of human fibroblasts on CHAP based scaffold^34^. Developing a multifunctional scaffold, which combines excellent biocompatibility, osteoinductivity, and antibacterial ability, which is considered to be the next-generation orthopedic implants for hard tissue engineering applications^43,44^. Selenium, silver, and antibacterial drugs, such as cephalexin and chlorhexidine, were commonly used to impart antibacterial function to the composite scaffolds^45–48^. Nguyen et al*.* investigated that selenium nanoparticles could inhibit *Staphylococcus aureus*, with low toxicity to mammalian cells^49^. Wang et al. identified that after coating poly(ether ether ketone) (PEEK) medical devices with selenium nanoparticles, the growth of *Pseudomonas aeruginosa* could be significantly inhibited^29^. However, the uncontrolled release of drugs, the potential systemic toxicity of medicine, and the aggregation of nanoparticles hindered the potential functions of the scaffolds. For these reasons, drugs with selenium, and silver were mainly encapsulated, or mixed, coated into the scaffold^50,51^. Here, we first fabricated the selenium-doped biphasic calcium phosphate nanoparticles, which were incorporated with silver nanoparticles.

The physicochemical properties of the obtained _Ag_SeB-NPs were deeply characterized by FT-IR, XRD, UV–Vis, Raman, and XPS analysis. In this study, SeB-NPs were synthesized by co-precipitation and ion-exchange sorption, aka, post-precipitation, occurred simultaneously. By substituting PO_4_^3−^ on the surface of BCP-NPs to adsorb SeO_3_^2−^ group, then a port of SeO_3_^2−^ ions entered the lattice (HA or β-TCP)^52^. The addition of selenium dopant could influence the phase composition, and the resulting SeB-NPs could be tentatively represented as Ca_10_(PO_4_)_X_(SeO_3_)_(6-X)_(OH)_2_ + Ca_3_(PO_4_)_X_(SeO_3_)_(6-X)_. The FT-IR spectra of SeB-NPs and BCP-NPs showed the *v*_1_ + *v*_3_ phosphate vibration in the region of 1,200–900 cm^−1^ and *v*_4_ phosphate bands in the range of 650–500 cm^−1^, as shown in Fig. 2a^53^. The O-Se-O asymmetric bond stretching at 731 cm^−1^ was detected for SeB-NPs, and the SeO_3_^2−^ group at 783 cm^−1^ was measured as well. With decreased Ca/Se mole ratio, the peaks belonging to the SeO_3_^2−^ group became stronger, which confirmed the presence of SeO_3_^2−^ group in SeB-NPs^54^. After XPS analysis of SeB-NPs, there was one peak at 58 eV of the Se3d scanning, which also proved that only SeO_3_^2−^ [Se (IV)] instead of SeO_4_^3−^ [Se (VI)] was incorporated into SeB-NPs (Fig. 5).

However, after the deposition of silver nanoparticles on the surface of SeB-NPs, no chemical bonds were formed between silver and SeB-NPs (Figs. 2, 3). Next, the crystallinity of SeB-NPs and _Ag_SeB-NPs was evaluated by XRD, as shown in Fig. 4a. The appearance of HA and β-TCP in all samples was expected. The relative intensity of the diffraction peak at 25.9° (0 0 2) (*D*_(002)_, Miller’s plane) was chosen to calculate the crystallite size (Scherrer equation)^55,56^. The crystallite means size varied for BCP-NPs, SeB-NPs, and _Ag_SeB-NPs, were calculated by Scherrer equation (Supplementary Materials). Compared with BCP-NPs, the unit cell dimensions of SeB-NPs were decreased, which proved the incorporation of SeO_3_^2−^ group. This inclusion was due to the different shape between SeO_3_^2−^ group (trigonal pyramids) and PO_4_^3−^ group (tetrahedra), and phosphate-to-selenite substitution resulted in paired Ca^2+^ and OH^−^ vacancies to rebalance the charge^57^. As the Ca/Se mole ratio decreased, the corresponding crystallinity increased. However, the crystallinity of _Ag_SeB-NPs with different AgNO_3_ concentrations changed slightly, mainly because silver nanoparticles were only deposited on the surface of SeB-NPs without chemical reaction occurred, which was consistent with the UV–Vis DRS testing results. Rameshbabu et al. identified that silver could influence the crystallinity of HA via heat treatment and further changed the nanosize of HA^55^. Furthermore, the morphology of SeB-NPs showed a typical needle-like bundle shape, which was different from BCP-NPs (Fig. 6 and Figure S1, Supplementary Materials). Unfortunately, the strong tendency to agglomerate made it impossible to evaluate its size distribution. After the deposition of silver, the morphology of _Ag_SeB-NPs tended to be further aggregated, and _Ag_SeB-NPs changed to snowflake-like agglomerate with increasing AgNO_3_ concentration. Element mapping analysis confirmed that selenium was uniformly distributed in SeB-NPs, and silver was spread on the surface of _Ag_SeB-NPs (Figs. 6 and 7).

Selenium has been widely applied in diverse areas such as the food and pharmaceutical industries. Many studies have confirmed that selenium doped HA not only exhibited low cytotoxicity for osteoblastic cells but also reduced the chance of tumor recurrence^24,29^. It was necessary to estimate the toxicity of the obtained SeB-NPs and _Ag_SeB-NPs for further tissue engineering applications. Osteoblasts were commonly used to evaluate the cytocompatibility of nanoparticles and/or scaffold in tissue engineering^58,59^. In this study, the cytocompatibility of SeB-NPs and _Ag_SeB-NPs was assessed by culturing with hFOB 1.19 cells. BCP-NPs possessed excellent cytocompatibility, and the incorporation of selenium into BCP-NPs did not reduce the cellular biocompatibility^60^. According to CCK-8 analysis (Fig. 8), osteoblasts proliferated with SeB-NPs over days, and the growth of cells was not influenced by the Ca/Se mole ratio. After the deposition of silver, cells cultured with all _Ag_SeB-NPs samples had similar proliferation profiles compared with SeB-NPs. Not only can the addition of selenium and silver improve the growth of the cells with BCP-NPs, but the antibacterial activity was acquired, which has potential for infection-resistant replacement materials^61,62^.

Finally, the typical Gram-positive *S. aureus* and Gram-negative *E. coli* bacteria were used to evaluate the antibacterial activity of synthesized SeB-NPs and _Ag_SeB-NPs. *S. aureus*, the most common virulent pathogen, and due to the biofilm formation and documented antibiotic resistance, can cause bone-implants-associated infections in hospitalized patients. Here, *S. aureus* was used together with *E. coli* (due to the highly capable of reduction)^63^. Selenium could promote the formation of superoxide radicals and, enhance oxidative stress, which resulted in the damage of bacterial cell walls^64^ Besides, selenium could inhibit the biofilm formation of the *S. aureus* and the growth of *E. coli*^65,66^. Figures 9 and 10 illustrated the antibacterial activity of SeB-NPs and _Ag_SeB-NPs against *S. aureus* and *E. coli* colonies. It was noted that the introduction of selenium in BCP-NPs could not empower the antibacterial performance on SeB-NPs. Besides, the antibacterial activity of the selenium-doped hydroxyapatite nanoparticles (SeHA-NPs) was investigated herein for comparison (Figures S9 and S10, Supplementary Materials). The results revealed that SeHA-NPs didn’t display inhibitory activity against *S. aureus* and *E. coli*, which was slightly different from previous reports^58,60^. The different results might be due to the varying morphology of SeHA-NPs used in this work, demonstrating that the antibacterial effect of nanoparticles is greatly influenced by the shape and size.

**Figure 11:**
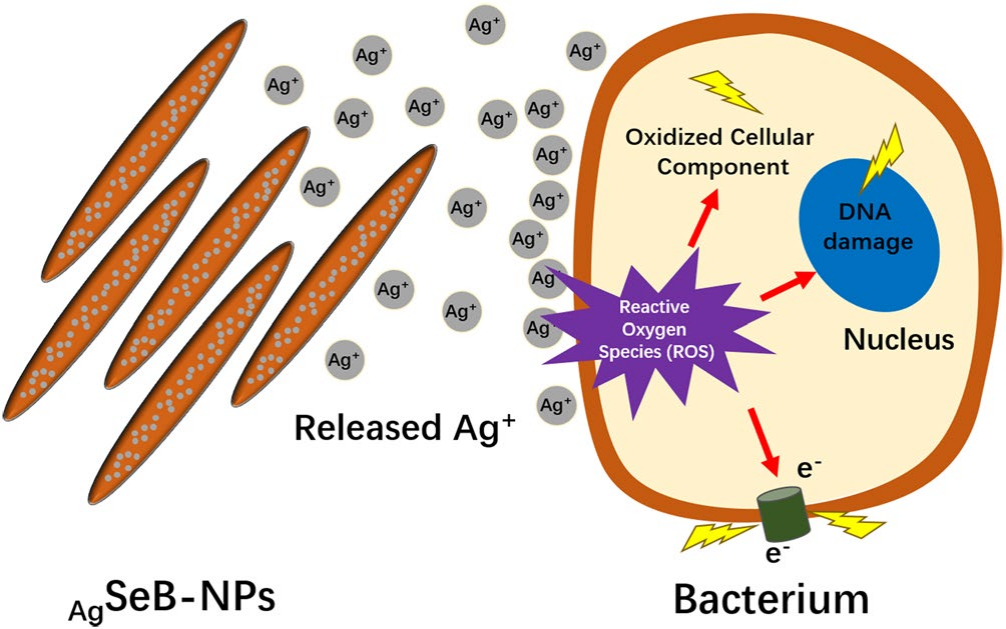
Mechanism of antibacterial activity of prepared _Ag_SeB-NPs against bacteria. Silver ions were released from _Ag_SeB-NPs to produce free radicals, which resulted in reactive oxygen species (ROS), and damaged bacteria until bacterial death.

However, after deposition of silver on SeB-NPs, the growth of *S. aureus* and *E. coli* were both decreased significantly. Assuming that a large number of silver nanoparticles might be released from _Ag_SeB-NPs, which can inhibit the initial bacterial adhesion and growth, the mechanism of prepared _Ag_SeB-NPs was displayed in Fig. 11. Previous papers reported that the release of silver nanoparticles could be regulated by pH, calcium, and phosphate ion concentrations in the surrounding medium^67^. Notably, the acidic environment could generate the release of silver nanoparticles. Therefore, because of the growth of oral bacteria under acidic conditions, the future use of _Ag_SeB-NPs could be expanded to dental applications^68^. Besides, selenium-doped bone mineral nanoparticles performed effective bone tumor inhibition. Thus, _Ag_SeB-NPs has potential advantages in the fabrication of a multifunctional bone scaffold for healing related bone tumor diseases. The balance between biocompatibility and antibacterial properties of _Ag_SeB-NPs, i.e., the excellent cytocompatibility, and productive inhibition activity against bacteria, supported the significance of _Ag_SeB-NPs in biomedical applications. However, future applications require further in vitro and in vivo investigations, including the release profile of silver nanoparticles and selenium.

## Conclusions

In summary, a well-developed nanostructured SeB-NPs and _Ag_SeB-NPs with excellent biocompatibility and suitable antibacterial properties were fabricated herein. FI-IR, Raman, XRD and XPS analysis certified that the SeO_3_^2−^ was doped at PO_4_^3−^ position. The needle-cluster-like morphology of SeB-NPs was obtained, and the formed _Ag_SeB-NPs tended to flock together, which was confirmed by TEM. After culturing with hFOB 1.19 cells, both SeB-NPs and _Ag_SeB-NPs behaved excellent cytocompatibility. Next, the Gram-negative *E. coli* and Gram-positive *S. aureus* were used to verify that _Ag_SeB-NPs had better antibacterial activity than SeB-NPs. The results obtained support the significance of _Ag_SeB-NPs in diverse biomedical applications.

## Supplementary information

Supplementary information

## Data Availability

All data generated or analyzed during this study are included in the published article and its Supplementary Materials files and are available from the corresponding author on reasonable request.
