# Supplementary material for: Nanostructured selenium-doped biphasic calcium phosphate with in situ incorporation of silver for antibacterial applications
Source: Sci Rep. 2020 Aug 13;10:13738. doi: 10.1038/s41598-020-70776-7 (PMC7427101; doi:10.1038/s41598-020-70776-7)
Supplement: Supplementary file 1 — Supplementary information [file 41598_2020_70776_MOESM1_ESM.docx]

Supplementary Materials

Nanostructured Selenium-doped Biphasic Calcium Phosphate with *In situ* Incorporation of Silver for Antibacterial Applications

Lei Nie^1,2,^*, Mengjuan Hou^1^, Tianwen Wang^1^, Meng Sun^1^, Ruixia Hou^3^

^1^ College of Life Sciences, Xinyang Normal University, Xinyang 464000, China

^2^ Department of Mechanical Engineering, Member of Flanders Make, KU Leuven (Catholic University of Leuven), Leuven 3001, Belgium

^3^ Medical School of Ningbo University, Ningbo 315211, China

* Corresponding author: Lei Nie
Post address: College of Life Sciences, Xinyang Normal University (XYNU), Xinyang 464000, P. R. China.
Tel: +86-13600621068
E-mail address: nielei@xynu.edu.cn; nieleifu@yahoo.com

ORC iD: 0000-0002-6175-5883

Materials and Methods

**Preparation of Selenium-doped Hydroxyapatite Nanoparticles (SeHA-NPs)**

The SeHA-NPs were prepared according to the previous report with further modification [1]. Briefly, 50 mL mixed solution of (NH_4_)_2_HPO_4_ and Na_2_SeO_3_ into 50 mL solution of Ca(NO_3_)_2_·4H_2_O (1.695 M) were added in a three-neck flask (the molar ratio of Ca/(P + Se) is 1.55, and the molar ratio of Ca/Se is 0.15), and the pH was adjusted to 11.0 by adding ammonium solution during the synthesis. The mixed solution was stirred for 24 h, and the precipitates were collected by centrifugation (10 000 rpm) and washed using Millipore water five times. The obtained nanoparticle was represented as SeHA 1, once the Ca/Se was adjusted to 0.30, the obtained nanoparticles was represented as SeHA 2.

**CCK-8 Assay**

The CCK-8 assay was used to evaluate the cytocompatibility of prepared SeB-NPs and _Ag_SeB-NPs by culturing with hFOB 1.19 cells. After removal of the culture media from cell culture plates, 300 μL fresh culture media and 30 μL CCK-8 kit solutions were immediately added and homogeneously mixed and then incubated for 4 h in a CO_2_ incubator. Finally, 200 μL reaction solutions were put into a 96-well plate. The optical density of each well at 450 nm was read by a microplate reader (SpectraMax 190, Molecular Devices, USA).

**The Crystallite Means Size Calculation**

After the phase composition was tested by using XRD and X’pert Pro Diffractometer (Phillips), the crystallite means size of prepared SeB-NPs and _Ag_SeB-NPs could be calculated using Scherrer’s formulation as follow:

$$D= \frac{0.94 \times\lambda}{\beta_{1/2} \times cos\theta^{'}}$$

Where D is the crystallite size in nanometers, λ is the wavelength of radiation in nanometers, *β_1/2_* is the full peak width at half maximum, and θ is the diffraction angle of the corresponding reflex.

**Storage of SeB-NPs and _Ag_SeB-NPs**

After the SeB-NPs and _Ag_SeB-NPs were obtained, the microparticles were freeze-dried at -55 °C for 48 h to get the powder and stored at RT under nitrogen atmosphere. Besides, the obtained SeB-NPs and _Ag_SeB-NPs could also be dispersed in Millipore water and stored as microparticles solution at 2-8 °C.

**The concentration of SeB-NPs and _Ag_SeB-NPs Testing**

Once the obtained SeB-NPs and _Ag_SeB-NPs were dispersed in Millipore water and stored as microparticles solution at 2-8 °C. The concentration will be further tested again before use. 100 μL nanoparticles solution was dropped on Aluminium foil, and the used Aluminium foil was weighed as *M_1_* (g). Then nanoparticles solution on Aluminium foil was dried, then the dried microparticles with Aluminium foil was weighted as *M_2_* (g); finally, the nanoparticles concentration (μg mL^-1^) was given as:

$$C=\frac{(M_{2}-M_{1})\times{10}^{6}}{100 \times{10}^{-3}}$$

**Table S1.** The molar concentration of Ca(NO_3_)_2_·4H_2_O, (NH_4_)_2_HPO_4,_ and Na_2_SeO_3_ varied for fabricating selenium-doped biphasic calcium phosphate nanoparticles.

| **Nanoparticles** | **SeB1** | **SeB2** | **SeB3** |
| --- | --- | --- | --- |
| Ca(NO_3_)_2_·4H_2_O | 1.69 | 1.69 | 1.69 |
| (NH_4_)_2_HPO_4_ | 1.35 | 0.92 | 0.82 |
| Na_2_SeO_3_ | 0.07 | 0.14 | 0.24 |


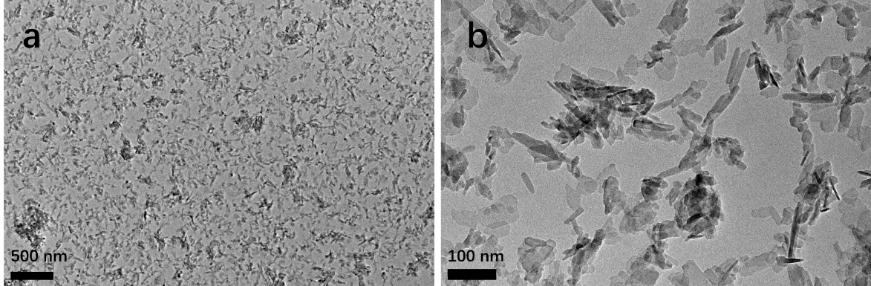


**Figure S1.** TEM images of biphasic calcium phosphate nanoparticles (BCP-NPs) at different magnifications.


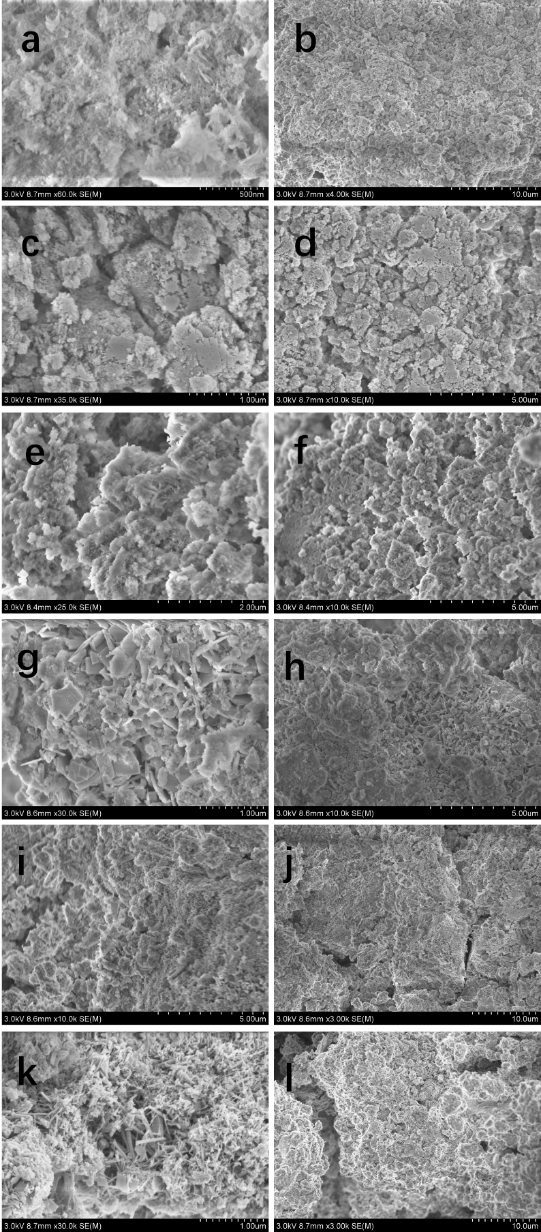


**Figure S2.** SEM images of SeB-NPs and _Ag_SeB-NPs at different magnifications. (**a**) SeB1, (**b**) SeB2, (**c**) SeB3, (**d**) _Ag_SeB1, (**e**) _Ag_SeB2, (**f**) _Ag_SeB3.


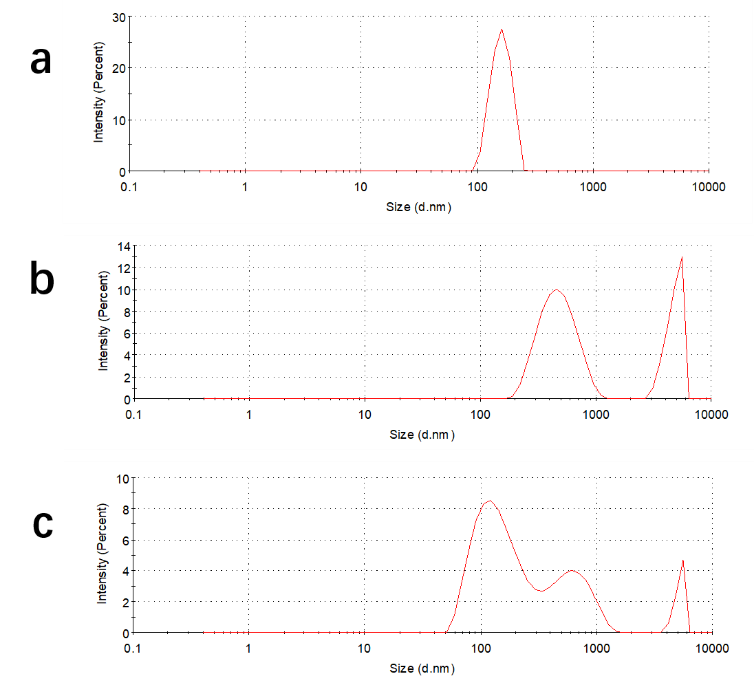


**Figure S3.** DLS analysis of (**a**) BCP-NPs, (**b**) SeB-NPs (SeB2), and (**c**) _Ag_SeB-NPs (_Ag_SeB2).


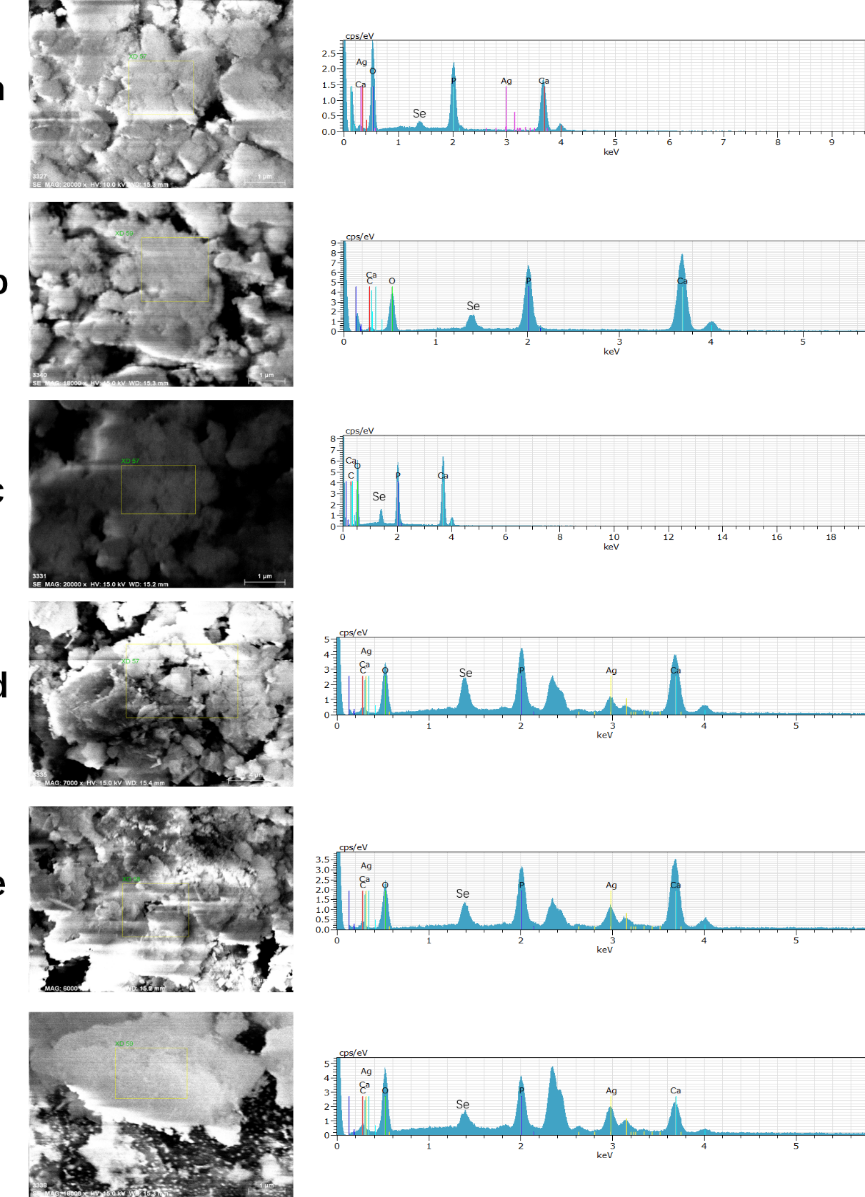


**Figure S4.** EDX analysis of SeB-NPs and _Ag_SeB-NPs. (**a**) SeB1, (**b**) SeB2, (**c**) SeB3, (**d**) _Ag_SeB1, (**e**) _Ag_SeB2, (**f**) _Ag_SeB3. SEM images of nanoparticles were shown first, then the region in rectangle was analyzed with EDX spectra.


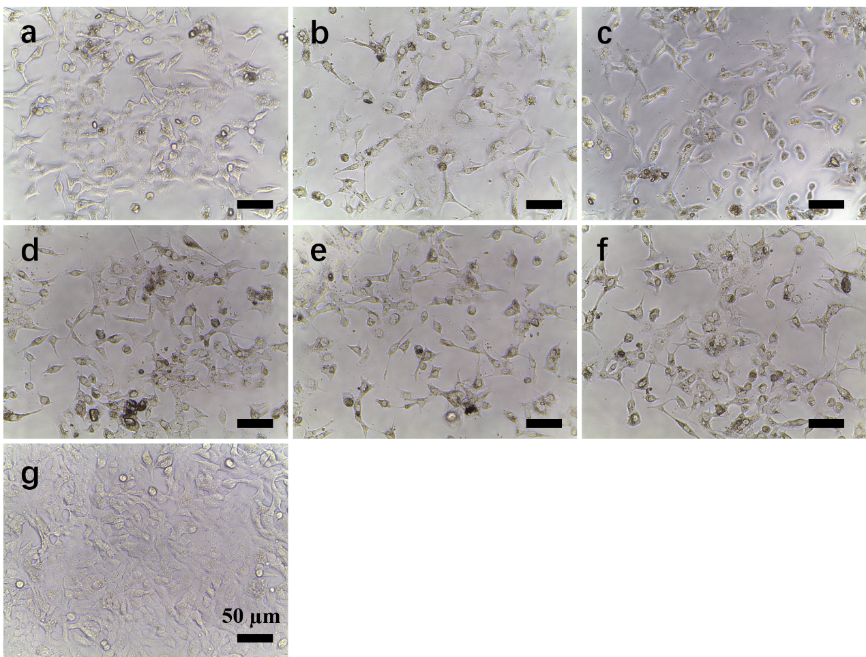


**Figure S5.** Optical microscopy images of hFOB 1.19 cells after culturing with SeB-NPs and _Ag_SeB-NPs for 3 days. (**a**) SeB1, (**b**) SeB2, (**c**) SeB3, (**d**) _Ag_SeB1, (**e**) _Ag_SeB2, (**f**) _Ag_SeB3, (**g**) control group. The nanoparticle concentration for all samples is 2000 µg/mL.


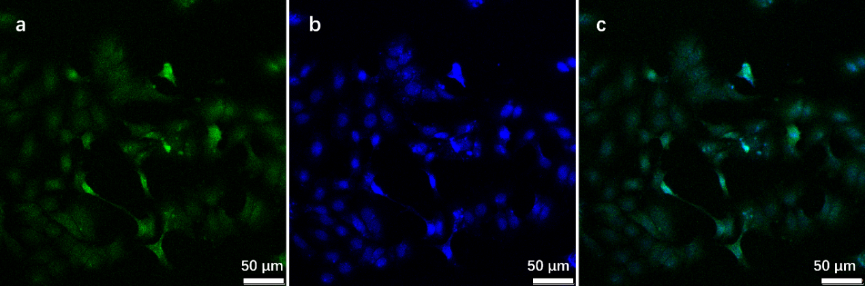


**Figure S6.** Fluorescent images of hFOB 1.19 cells after culturing with _Ag_SeB-NPs (_Ag_SeB3) on day 3, (**a**) FITC-Phalloidin, (**b**) DAPI, and (**c**) Merged. The nanoparticle concentration is 2000 µg/mL.


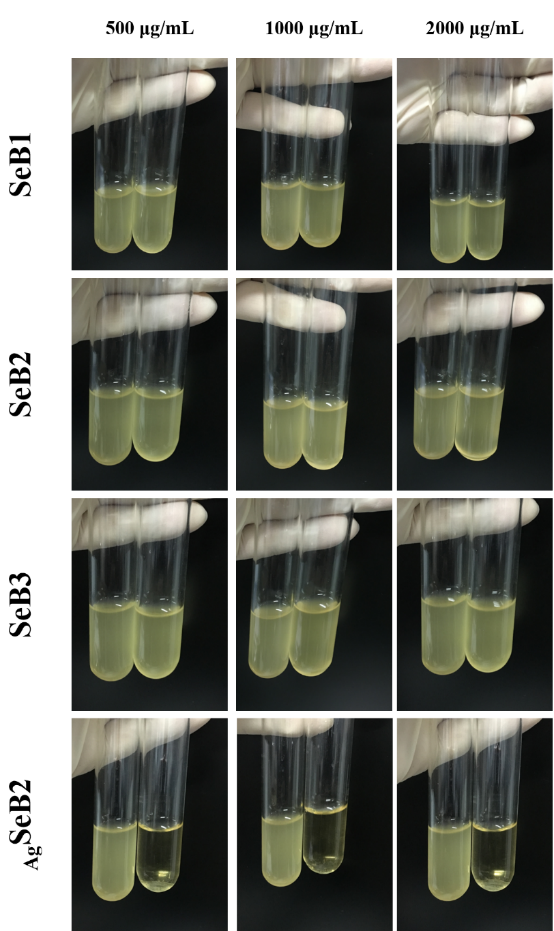


**Figure S7.** Photos of *E. coli* and *S. aureus* grown in the actual culture tubes after the addition of SeB-NPs and _Ag_SB-NPs after 12 h.


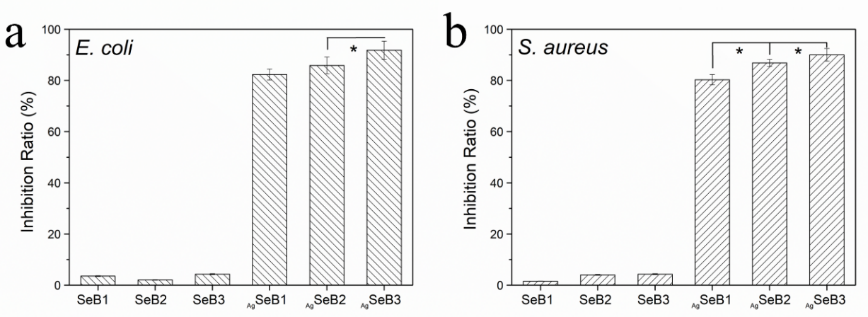


**Figure S8.** The inhibition ratio of *E. coli* and *S. aureus* grown o in the actual culture tubes after the addition of SeB-NPs and _Ag_SB-NPs after 12 h.


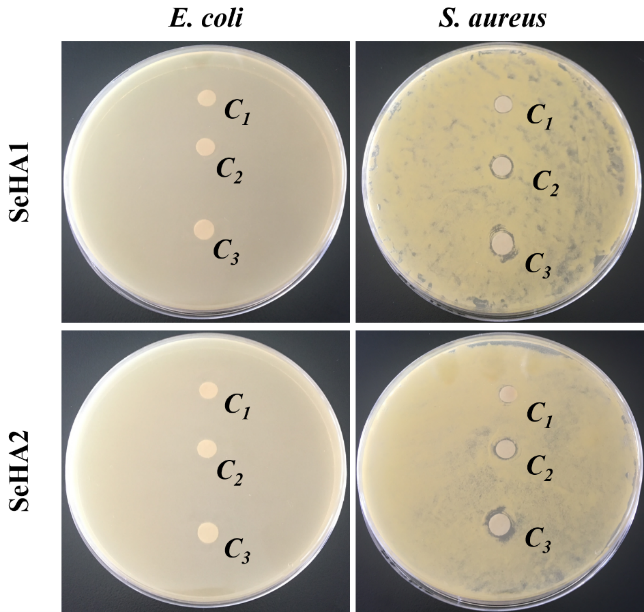


**Figure S9.** Photos of *E. coli* and *S. aureus* grown on nutrient agar LB plates after the addition of selenium-doped hydroxyapatite (SeHA-NPs) after 12 hours.


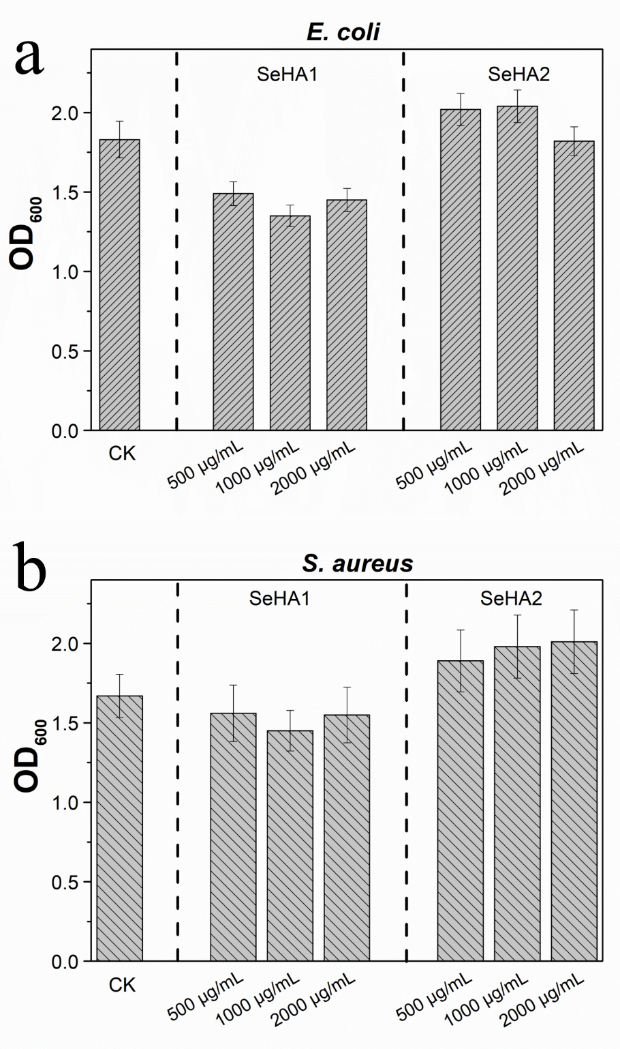


**Figure S10.** The inhibition ratio of *E. coli* and *S. aureus* grown on nutrient agar LB plates after the addition of selenium-doped hydroxyapatite (SeHA-NPs) after 12 hours.

References

1. Wang, Y. *et al.* In vitro and in vivo mechanism of bone tumor inhibition by selenium-doped bone mineral nanoparticles. *ACS Nano.* **10**, 9927-9937 (2016).
2. Zhou, Q. et al. Synthesis and characterization of silver nanoparticles-doped hydroxyapatite/alginate microparticles with promising cytocompatibility and antibacterial properties. Colloids and Surfaces A: Physicochemical and Engineering Aspects 585, 124081 (2020).
